# Technological Functionalisation of Microencapsulated Genistein and Daidzein Delivery Systems Soluble in the Stomach and Intestines

**DOI:** 10.3390/pharmaceutics16040530

**Published:** 2024-04-12

**Authors:** Jurga Andreja Kazlauskaite, Inga Matulyte, Mindaugas Marksa, Jurga Bernatoniene

**Affiliations:** 1 Department of Drug Technology and Social Pharmacy, Lithuanian University of Health Sciences, LT-50161 Kaunas, Lithuania; jurga.andreja.kazlauskaite@lsmu.lt (J.A.K.); inga.matulyte@lsmu.lt (I.M.); 2 Institute of Pharmaceutical Technologies, Lithuanian University of Health Sciences, LT-50161 Kaunas, Lithuania; 3 Department of Analytical and Toxicological Chemistry, Lithuanian University of Health Sciences, LT-50161 Kaunas, Lithuania; mindaugas.marksa@lsmu.lt

**Keywords:** microcapsules, drug delivery, natural compounds, isoflavones, red clover, chitosan, sodium alginate

## Abstract

Encapsulating antioxidant-rich plant extracts, such as those found in red clover, within microcapsules helps protect them from degradation, thus improving stability, shelf life, and effectiveness. This study aimed to develop a microencapsulation delivery system using chitosan and alginate for microcapsules that dissolve in both the stomach and intestines, with the use of natural and synthetic emulsifiers. The microcapsules were formed using the extrusion method and employing alginate or chitosan as shell-forming material. In this study, all selected emulsifiers formed Pickering (β-CD) and traditional (white mustard extract, polysorbate 80) stable emulsions. Alginate-based emulsions resulted in microemulsions, while chitosan-based emulsions formed macroemulsions, distinguishable by oil droplet size. Although chitosan formulations with higher red clover extract (C1) concentrations showed potential, they exhibited slightly reduced firmness compared to other formulations (C2). Additionally, both alginate and chitosan formulations containing β-CD released bioactive compounds more effectively. The combined use of alginate and chitosan microcapsules in a single pill offers an innovative way to ensure dual solubility in both stomach and intestinal environments, increasing versatility for biomedical and pharmaceutical applications.

## 1. Introduction

Various medicinal applications have been attributed to isolated natural bioactive substances, including pure phytochemicals, botanical extracts, and essential oils. Among these, red clover stands out for its phenolic content, especially isoflavones, which are compounds with estrogenic properties. Isoflavones exhibit promise in addressing diverse menopausal symptoms like hot flashes, supporting cardiovascular health, and combating osteoporosis [1,2,3].

Natural compounds, such as phenolic compounds, offer beneficial properties like antioxidants, antimicrobial, and anti-inflammatory effects, which are valuable for various applications. However, they are susceptible to environmental factors such as heat and light, have low water solubility, undergo rapid metabolism, and are quickly eliminated from the body. Additionally, they can degrade in water, undergo oxidation, and lose activity over time [4,5]. Encapsulating antioxidant-rich plant extracts within microcapsules safeguards them from degradation, boosting stability, shelf life, and efficacy. Additionally, plant-derived compounds demonstrate excellent solubility in both water and oil phases, facilitating the creation of stable, homogeneous emulsions with uniform dispersion of natural components, crucial for desired functionalities [6]. Recently, there has been an extensive interest in research focusing on emulsions stabilised by natural compounds [7]. In this study, emulsifiers and polymers known for their stability-enhancing and solubilising properties of bioactive compounds were carefully selected. Mustard extract, β-cyclodextrin (β-CD), and polysorbate 80 have been chosen for their emulsifying capabilities.

The white mustard extract plays a crucial role in forming traditional oil-in-water emulsions. This extract contains lecithin, the primary emulsifier in egg yolks, contributing to emulsion stability [8]. Studies have demonstrated that mustard powder can also effectively stabilise emulsions, with research showing stability for up to one month when stabilised with mustard or cinnamon. Additionally, mustard extract’s capacity to absorb and retain liquid further enhances its effectiveness as an emulsifying agent [9,10]

β-CD can enhance emulsion stability and antioxidant properties when combined with conventional emulsifiers. Its ability to bring antioxidant-active substances to the oil-water interface makes it valuable in emulsions seeking antioxidant effects [11,12]. Additionally, β-CD forms inclusion complexes with compounds, allowing for targeted ingredient delivery. Emulsions with β-CD as an emulsifier are Pickering emulsions, offering better stability and droplet size control [13]. Pickering emulsion differs from traditional emulsions as solid particles rather than surfactants stabilise it. These solid particles, typically amphiphilic, possess both hydrophilic and hydrophobic traits, allowing them to attach to the oil-water interface and uphold the stability of the emulsion [14].

Polysorbate 80 is a synthetic non-ionic surfactant widely used as an emulsifier in emulsions. It has several functions, including emulsification, dispersion, wetting and stabilisation. It is also known for its solubilising properties, allowing it to enhance the solubility of poorly soluble substances. It acts as an emulsifier in pharmaceutical formulations, ensuring the uniform distribution of active ingredients and improving their stability. This makes it useful in various oral, parenteral, and topical formulations where increased solubility is desired [15,16,17]. Due to its viscous nature, it contributes to the texture and stability of products.

Besides overcoming stability problems, microencapsulation of emulsions reduces the perception of possible off-flavours or colours from the encapsulated material, facilitates storage, and extends shelf life without adverse influence on the physical, chemical or functional properties [18]. Coacervation, a widely used encapsulation technique for probiotics, involves the formation of a liquid rich in polymer phase in equilibrium with another liquid phase. This colloidal phenomenon can manifest in either a low or a highly dispersed state, offering promising encapsulation capabilities. There are various microencapsulation techniques employing coacervation, including spray drying, freeze drying, extrusion, spray cooling/chilling, fluidised bed drying, and more [6,19]. Extrusion is a widely used encapsulation technology for encapsulating different materials, microorganisms and phenols in hydrocolloid gel matrices [19,20].

Polymers such as chitosan and alginate, alongside dendrimers and others, have emerged as common choices for encapsulation matrices. These polymers offer distinct advantages in terms of biocompatibility, biodegradability, and functionality, making them well-suited for encapsulating a diverse array of bioactive compounds [21].

Sodium alginate, derived from brown seaweed, is a versatile anionic polymer renowned for its biocompatibility and low toxicity [22]. Its mild gelation upon exposure to divalent cations has spurred extensive exploration in biomedical fields. Alginate hydrogels, formed through diverse cross-linking techniques, mimic the structural properties of living tissue extracellular matrices, making them valuable for wound healing, bioactive agent delivery, and cell transplantation applications [23]. It also serves various beneficial roles in food products, such as gelling, thickening, and coating. Additionally, as a polyelectrolyte, it contributes to controlling solution rheology through external stimuli like pH and ionic strength. This property holds promise for manipulating emulsion rheology, showcasing practical applications in the field [24,25]. 

Chitosan-based particles are highly regarded as ideal drug carriers because of their biodegradable, biocompatible, and non-toxic nature. Additionally, the abundance of functional amino and hydroxyl groups in chitosan allows for the efficient immobilisation of active substances through chemical interactions [26]. The pH-responsive properties of chitosan, stemming from its amino groups and sodium alginate from its carboxyl groups, significantly enhance its suitability as a drug carrier. Leveraging these attributes, microencapsulation via extrusion techniques utilising both polymers proves to be a promising approach for drug delivery applications [27,28].

This research focuses on microencapsulation, which revolves around the careful selection of natural emulsifiers and polymers. Notably, the study emphasises the application of mustard extract, β-cyclodextrin, and polysorbate 80 to stabilise and solubilise bioactive compounds such as genistein and daidzein sourced from red clover extract. This combination of natural compounds represents a novel approach that enhances the encapsulation process, ensuring the stability and bioavailability of fragile bioactive molecules. This research addresses existing challenges in bioactive delivery and also paves the way for targeted delivery applications in pharmaceuticals, nutraceuticals, and functional foods from red clover extract. Therefore, the study aims to develop and optimise a microencapsulation delivery system, employing chitosan and alginate for stomach- and intestine-soluble microcapsules, respectively, using different emulsifiers and evaluating their stability, physical parameters and in vitro release profiles for genistein and daidzein in simulated gastrointestinal conditions.

## 2. Materials and Methods

### 2.1. Plant Material and Reagents

*Trifolium pratense* L. plant flower buds were collected in *Trifolium pratense* L. fields, Kupiškis district, Lithuania. The mustard powder was purchased at SALDVA. Ethanol (96%) used for extraction was purchased from Vilniaus degtine. (Vilnius, Lithuania). In this experiment, purified water was prepared with GFL2004 (GFL, Burgwedelis, Germany). Deionised water was prepared with Millipore, SimPak 1 (Merck, Darmstadt, Germany). 

The following reagents were used as standards: genistein, daidzein, and biochanin A (Sigma-Aldrich, Steinheim, Germany). β-CDs were purchased from Sigma-Aldrich (Hamburg, Germany); Folin–Ciocalteu’s phenol reagent (Merck, Darmstadt, Germany); shell material, alginic acid sodium salt from brown algae obtained from Sigma-Aldrich (Shanghai, China) was used. Calcium chloride (Farmalabor, Pozzillo, Italy) salt formulated microcapsules as a crosslinker. Medium molecular weight (MMW) chitosan, high molecular weight (HMW) 80/1000 chitosan, and high molecular weight (HMW) 80/3000 chitosan were purchased from Sigma-Aldrich (Steinheim, Germany). Emulsifiers Tween 80^®^ were purchased from Sigma-Aldrich (Germany). Acetic acid and sodium hydroxide were obtained from Sigma-Aldrich (Germany).

#### 2.1.1. Preparation of *Trifolium pratense* L. (Red Clover) Extract

The extract was prepared following the method outlined in a previous study [29]. The method used 1% of the excipient β-CD in a solution of 50% ethanol/water. Ultrasound-assisted extraction was conducted using a Grant Instruments™ XUB12 Digital ultrasound bath (Cambridge, UK) operating at 38 kHz for 10 min at a temperature of 40 ± 2 °C. Afterwards, the samples were refluxed for 1 h. The mixture was then cooled down, followed by centrifugation at 3382× *g* (5500 rpm) for 10 min, and the supernatant was concentrated in a porcelain dish.

#### 2.1.2. Preparation of *Sinapis alba* (White Mustard) Extract

White mustard extraction was conducted using a Grant Instruments™ XUB12 Digital ultrasound bath (Cambridge, UK) operating at 38 kHz. 10.0 ± 0.001 g of dried and milled powder was macerated in 50 mL of water. Ultrasound processing was performed for 30 min at a temperature of 40 ± 2 °C. After processing, the samples were centrifuged at 3382× *g* (5500 rpm) for 10 min, and the supernatant was decanted. The extract was then filtered through a paper filter for subsequent use in the research.

### 2.2. Emulsion Preparation

#### 2.2.1. Alginate Solution Preparation

Initially, a 2% sodium alginate solution was prepared by dissolving alginic acid sodium salt in distilled water until complete solubilisation. This solution, serving as the shell material soluble in gut media, was utilised for emulsion preparation throughout the experiment. Two concentrations of alginate solution, namely 0.5% and 2%, were employed in the study.

#### 2.2.2. Chitosan Solution Preparation

Various chitosan gels were prepared using different concentrations and molecular weights, including 2% MMW chitosan gel, 4% MMW chitosan gel, 2% HMW 80/1000 chitosan gel, and 2% HMW 80/3000 chitosan gel.

Chitosan powder was gradually added to acetic acid solution at concentrations ranging from 1% to 4% to form the gels. Stirring with an IKA EURO-STAR 200 digital stirrer (Staufen, Germany) ensued until a clear gel was achieved. The gel was centrifuged for 2 min at 447× *g* (2000 rpm) in 50 mL centrifuge tubes using a Sigma 3-18KS centrifuge (GmbH, Osterode am Harz, Germany) to remove air bubbles. Microcapsules were formed from the chitosan solution with ethanolic extract alone, employing HMW 80/1000 chitosan at a minimum concentration of 2% acetic acid and 1% nitrogen hydroxide.

#### 2.2.3. Chitosan and Alginate Emulsions Preparation

To prepare the alginate emulsion with *Trifolium pratense* L. extract, a solution containing excipients (sweet almond oil, emulsifier-mustard extract, β-CD, or polysorbate 80-and xanthan) was combined with a sodium alginate solution. The mixture was stirred for 15 min using a magnetic stirrer (MSH-20A, Witeg, Wertheim, Germany), after which the extract was added. Formulations of the emulsions are detailed in Table 1.

Samples of chitosan and alginate emulsions were prepared using both ethanol and water extracts, but the ethanol extract is composed of more isoflavones and phenolic compounds, so only the ethanol extracts were used in further studies (Table 1 and Table 2).

The following research used 8 of the most stable emulsions. For this article, we will use the codes provided in Table 3 for easier compression. 

Before conducting any analysis on the emulsions (except the stability test), the samples are centrifuged at 112× *g* (1000 rpm) for 2 min. This step is to remove air bubbles, ensuring that the results are free from any interference caused by air.

### 2.3. Physical Parameters of Emulsions

#### 2.3.1. Emulsions Stability Determination

Emulsion stability was evaluated using a Sigma 3-18KS centrifuge (Sigma Laborzentrifugen GmbH, Osterode am Harz, Germany). The test, repeated three times, applied centrifugal forces from 1006× to 5478× *g* (3000 to 7000 rpm) for 5 min each. The test was conducted, and the percentage of non-layered emulsion, known as the centrifugation index (CI), was calculated using the following formula:

$$CI\;\left(\%\right)=\frac{Ve}{Vi}\cdot 100\%$$

where V_e_ represents the volume of the remaining emulsion after centrifugation, and V_i_ is the volume of the initial emulsion. The test was repeated three times, and the average CI was determined. Test parameters included a centrifugal force of 3000 rpm, a temperature of 23 °C, and a duration of 5 min.

#### 2.3.2. Particle Size and Distribution Measurements and Pictures

Oil particle size and distribution were analysed using the Mastersizer 3000 with a Hydro EV unit (Malvern Analytical Ltd., Malvern, UK). The emulsion was added drop-wise to water until laser obscuration reached 9.5–10.5%, maintaining a constant pump speed of 2400 rpm. Refractive indices of 1.330 and 1.478 were used. Average particle size distribution was calculated from five runs, with formulations characterised by percentile values (D10, D50, and D90).

For microscopic examination, samples were observed using a Nikon H550S microscope (Nikon, Chiyoda-ku, Japan) at 10× objective magnification. Images were captured with the NIS-Elements D3.2 program.

#### 2.3.3. Dynamic Viscosity

Emulsion viscosity was assessed using a Fungilab ALPHA series viscometer (Fungilab, Sant Feliu de Llobregat, Barcelona, Spain). Each measurement involved adding 50 mL of emulsion to the viscometer’s dedicated vessel and placing it on the apparatus’s surface. The L3 head was used, and it was set to 100 rpm. The viscosity (mPa·S) was recorded at room temperature. Each sample was measured until the value of dynamic viscosity was stable. 

### 2.4. Microcapsules’ Formulation and Preparation

Alginate microcapsules were prepared by extrusion method. Medical 10 mL syringes were placed in an NE-1000 Programmable Single Syringe Pump (KF T technology SRL, Rozzano, Italy) and used to form microcapsules. The drops of emulsion were ejected from the needle into the crosslinker solution. The height from the needle to the solution surface was 20 cm, and the pumping speed was 0.5 mL/min. A solution of 3% calcium chloride was used as a crosslinker. Microcapsules were prepared by using an MSH-20A magnetic stirrer at 150 rpm. Particles in the crosslinker solution were stirred for 15 min, and then the microcapsules were filtered using filter paper and washed with distilled water. Manufactured capsules were left to dry at room temperature for 24 h. Dried and wet microcapsules were stored in sealed tubes until further tests.

Chitosan microcapsules were prepared similarly to alginate microcapsules. Medical 10 mL syringes were placed in an NE-1000 Programmable Single Syringe Pump (KF T technology SRL, Rozzano, Italy) and used to form microcapsules. A 1% sodium hydroxide solution was used, and the magnetic stirrer MSH-20A was set at 130 rpm. Chitosan gel was slowly dropped from the syringe (13 cm, 0.2 mL/min) to the 1% sodium hydroxide solution. Particles in the cross-linker solution were stirred for 20 min, and then the microcapsules were filtered using filter paper and washed with distilled water. Manufactured capsules were left to dry at room temperature for 24 h. Dried and wet microcapsules were stored in sealed tubes until further tests.

### 2.5. Physical Parameters of Microcapsules

#### 2.5.1. Size and Shape of the Microcapsules

Microcapsule sizes were determined using a digital caliper (BGS Technic, Wermelskirchen, Germany). Thirty microcapsules were measured to calculate the mean and standard deviation of their diameters, both in dried and freshly prepared samples.

To examine morphological features, a light microscope Nikon H550S (Nikon, Chiyoda Ku, Japan) with an integrated monitor display was utilised. Emulsions and dried microcapsules were observed at 10× objective magnification using the NIS-Elements D3.2 program.

#### 2.5.2. Firmness of the Microcapsules

Microcapsule firmness was evaluated with the TA.XT.plus texture analyser (Texture Technologies, Brewster, NY, USA). Using a P/100 platen probe, the force required to compress a 2 mm microcapsule was measured on freshly prepared samples. The maximum force was set at 6500 g. Each sample was analysed with five microcapsules, and the mean and standard deviation were calculated from five measurements.

#### 2.5.3. Swelling Characteristic of Microcapsules

Dried microcapsules were swelled in water [30]. After 4 h, the microcapsules were weighed. Swollen microcapsules were then separated, filtered through a metal mesh, and dried with a paper towel to remove excess fluid. The swelling index (SI) was calculated using the following formula:

$$SI\;\left(\%\right)=\frac{Ws-Wi}{Wi}\cdot 100\%$$

where W_s_ is the weight of swollen microcapsules at the time, and W_i_ is the dried microcapsules’ weight.

### 2.6. Total Content of Active Compounds and In Vitro Release and Analysis of Microcapsules

#### 2.6.1. In Vitro Release of Active Compounds

The test used the Sotax AT7 Smart Dissolution System (SOTAX AG, Aesch, Switzerland). The gastric medium was prepared according to the European Pharmacopoeia. To prepare gastric juice, 2.0 g of NaCl, 80 mL of 1 M HCl solution, and 3.2 g of pepsin were combined with distilled water to reach a volume of 1000 mL (pH = 1.2). Simulated intestinal juice was made using 6.8 g of KH_2_PO_4_, 77.0 mL of 0.2 M NaOH solution, 10 g of pancreas powder, and distilled water up to 1000 mL. Samples in the gastric medium were incubated for 30–90 min before transferring to the intestinal medium for another 30–90 min. Sampling for HPLC analysis occurred every half hour within the total in vitro release time of 0–270 min.

For HPLC analysis, samples were filtered and prepared to detect isoflavones daidzein, genistein, and biochanin A.

#### 2.6.2. Total Phenolic and Flavanoid Content

The total phenolic content was determined as in our previous research [31]. Folin–Ciocalteu’s phenol reagent (1:9 dilution in distilled water) was used. A 0.5 mL sample was combined with 2.5 mL of 7% sodium carbonate. After mixing, the absorbance was measured at 765 nm after 1 h using a Shimadzu UV-1800 spectrophotometer (Kyoto, Japan). Gallic acid served for the calibration curve, with results expressed as mg GA/g dw gallic acid equivalents.

Flavonoid content was measured by mixing 0.1 mL of extract with 1.0 mL of 96% ethanol, 0.05 mL of 33% acetic acid, 0.15 mL of 10% aluminium chloride, and 2 mL of 5% hexaethylenetetraamine. After a 30-min reaction at a 475 nm wavelength, spectrophotometric analysis was conducted. Results were presented as rutin equivalents in mg RE/g dw, using a calibration curve from rutin standards.

#### 2.6.3. Isoflavones Determination Using High-Performance Liquid Chromatography

The release of daidzein, genistein and biochanin A were determined using high-performance liquid chromatography (HPLC) with diode array detectors (HPLC–PDA). The analysis was conducted using the Shimadzu Nexera X2 LC-30AD HPLC system (Shimadzu, Tokyo, Japan), equipped with an SPD-M20A diode array detector (DAD). The isoflavones’ research conditions are described in the previous work of Kazlauskaite et al. [32].

### 2.7. Statistical Analysis

Data were analysed using SSPS version 20.0 (IBM Corporation, Armonk, NY, USA). Physical parameters of emulsions and microcapsules (except for size) and total phenolic and flavonoid content experiments were performed three times. The size of microcapsules was performed using 30 microcapsules. Data are expressed as mean ± standard deviation (S.D.). The comparisons between three different measurements were made using Friedman and Wilcoxon tests. In addition, comparisons between the two groups were made using the Mann–Whitney U test. The results were considered statistically significant at *p* < 0.05.

## 3. Results and Discussion

### 3.1. Determination of Chitosan and Alginate Emulsions Parameters

#### 3.1.1. Emulsions Formation, Stability and Active Compounds Concentration

Various formulations of alginate emulsions were prepared, and their stability was assessed. In the case of alginate emulsions, variations in the concentration of additional water or extract did not significantly affect microcapsule formation. Consequently, our focus shifted towards investigating the impact of emulsifiers and alginate concentrations on both emulsions and microcapsules.

Conversely, when employing chitosan, the choice of extract proved to be influential in microcapsule formation. Notably, red clover extract altered the pH, hindering the formation of microcapsules at higher extract concentrations. Consequently, this investigation with chitosan prioritised assessing the effects of emulsifiers and the maximum extract concentration on both emulsion and microcapsule properties.

Optimal stability of alginitic samples was observed in formulations wherein extract concentrations remained below 35% (Table 1). The stability of these emulsions was systematically tested at three centrifugation speeds: 3000, 5000, and 7000 rpm. Following optimisation, eight preparations with alginate were identified as the most stable under centrifugation up to 5000 rpm. Formulations 3A and 4A notably exhibited stability, even at 7000 rpm.

**Figure 1:**
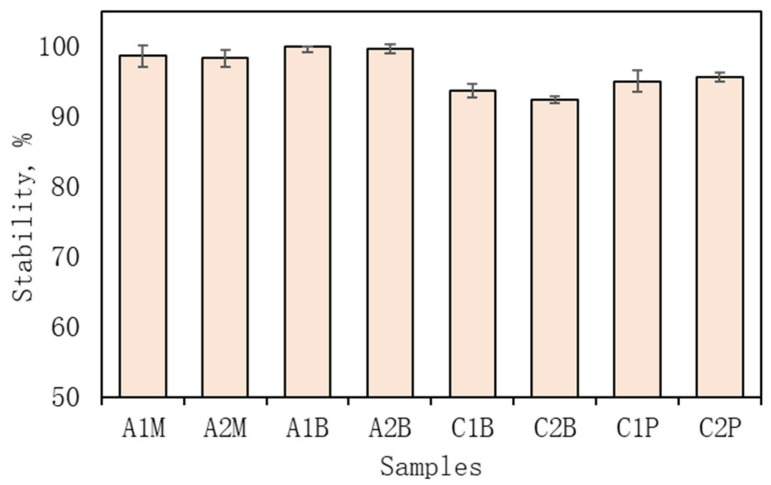
Stability at 7000 rpm on alginate and chitosan emulsions. The results are mean values (*n* = 3). Sample meanings are provided in Table 3.

Four samples were selected for further investigation into alginate emulsions: A1M, A2M, A1B, and A2B and their stability is provided in Figure 1. These emulsions featured varying concentrations of alginate solution (A1 and A2), specifically 0.5% (A2M, A2B) and 2% (A1M, A1B). The emulsifiers were mustard extract “M” and β-CD–“B” (Table 3). The stability of all alginate emulsion samples is provided in Figure 1.

In the study conducted by Wu et al., it was found that mustard extract outperformed gum arabic and citrus pectin in terms of both emulsion stability and surface activity among these three polysaccharides [33]. 

White mustard powder is rich in phytonutrients, particularly glucosinolates, and contains various minerals known for their anti-inflammatory properties, such as selenium and magnesium. Additionally, it serves as a good source of omega-3 fatty acids [34]. Mustard extract facilitates the formation of traditional emulsions and enhances the final product with supplementary advantages.

β-CD has extensive applications in the pharmaceutical industry. Its utilisation for preparing emulsions or enhancing their stability alongside conventional emulsifying agents has garnered increasing attention in research areas. Cyclodextrins’ capacity to facilitate emulsion formation has been extensively explored in the food and cosmetic industries. Studies comparing α-CD, β-CD, and γ-CD have revealed that β-CD exhibits the most pronounced emulsifying effect in the formation of Pickering emulsions [35,36].

In the alginate emulsions, xanthan gum was used as a co-emulsifier to enhance the stability of the system. In the literature, it was reported that higher xanthan gum concentrations ensure reaching the critical viscose concentration and complete retardation of creaming in emulsions [37]. 

In a parallel investigation, different concentrations and molecular weights of chitosan gels were prepared for emulsion formation, including 2% MMW chitosan gel, 4% MMW chitosan gel, 2% HMW 80/1000 chitosan gel, and 2% HMW 80/3000. Preliminary assessments determined that the chitosan solution could yield stable emulsions and sufficiently robust microcapsules. All chitosan gels were used with red clover ethanolic extract exclusively. Among them, 2% HMW 80/1000 was the sole chitosan solution that formed microcapsules at a minimum 2% acetic acid concentration. Consequently, all emulsions performed in this research were developed using this specific chitosan (HMW 80/1000).

Similarly to the alginate emulsions, stability evaluations were conducted at three centrifugation speeds: 3000, 5000, and 7000 rpm. While six formulations exhibited high stability, formulations 4C and 5C demonstrated remarkable stability even under centrifugation up to 7000 rpm, albeit not reaching 100% (Table 2). Four samples—C1P, C1B, C2P, and C2B—were chosen to investigate chitosan emulsions further. The chitosan concentration in samples was the same, but the amount of ethanolic red clover extract used differed between C1 (10%) and C2 (20%) (Table 3). Different emulsifiers in formulations were used where “P” signifies polysorbate 80 as the emulsifier (C1P and C2P) and “B” represents β-CD (C1B and C2B). The stability of all chitosan emulsion samples is provided in Figure 1.

In emulsions, chitosan acts not only as a shell material for later microcapsules but also as an emulsifier and emulsion stabiliser. It achieves this by forming a protective layer at the interfaces between oil and water, enhancing viscosity, and interacting with various surface-active agents such as surfactants, proteins, and polysaccharides [38]. Nevertheless, using higher concentrations of ethanolic red clover extract, such as in alginitic emulsions with chitosan, did not form stable emulsions or formed the emulsions, but was not enough to form robust microcapsules. Lowering chitosan concentration and increasing emulsifiers such as polysorbate 80 or β-CD did not yield the desired effect. Using xanthan gum to stabilise the emulsion system also did not help, and the emulsions were too thick for microcapsule formation. Therefore, the xanthan gum was removed from the formulation, and the chitosan concentration was increased. 

Polysorbate 80 stabilisation mechanism resides in the surfactant’s ability to reduce the interfacial tension by relatively short periods, and being a harmless surfactant contributes in more than one way to improve the efficacy of the drug in terms of increasing the solubility, bioavailability, and permeability of the drug [39,40].

Efforts have been dedicated to enhancing chitosan’s capability to transport hydrophobic substances, primarily through the functionalisation of its structure with cyclodextrins. In certain studies, researchers have combined the advantages of both carriers to engineer more efficient carrier systems. These innovations offer enhanced mucoadhesive properties for the effective release of drugs in biomedical applications, as well as improved protein delivery, sustained release of insecticides^,^ and removal of environmental contaminants. The results indicate that these carrier systems show promise as potential candidates for extending the longevity of sensitive or volatile compounds when exposed to environmental conditions [41,42]. 

In this research, after the preparation of stable emulsions, the total content of active compounds was determined in each sample (Table 4). Comparing the results of the emulsions with alginate, it is clear that the microcapsules with mustard would contain more bioactive ingredients. The mustard extract contains phenolic compounds, including flavonoids. The higher phenolic and flavonoid content was found in the A2M sample (Table 4). Nevertheless, the difference between the samples was statistically insignificant. The same situation was observed in samples A1B and A2B; even though sample A2B had higher bioactive ingredient concentrations, the differences were negligible in emulsions (Table 4).

In the formulations C1 and C2 of chitosan, the extract quantities used differ (C1—10% and C2—20%). Therefore, samples C2P and C2B had the highest bioactive compounds yields—C2B yielded 210.46 ± 11.56 µg/g daidzein, 36.21 ± 5.74 µg/g genistein and 24.54 ± 1.01 µg/g biochanin A; C2P consisted of 206.96 ± 12.87 µg/g daidzein 34.99 ± 6.98 µg/g genistein and 23.78 ± 2.64 µg/g biochanin A. The statistical difference of bioactive compounds between the formulations and emulsifiers was insignificant (Table 4).

The overall concentration of ethanolic red clover extracts in chitosan emulsions is lower compared to emulsions with alginate. Therefore, the results were lower in active compounds in all samples; the same concentrated ethanolic red clover extract was used.

The concentrated red clover extract was used in this research in order to have as high of an isoflavone concentration as possible. Red clover serves as the primary source for effectively isolating isoflavones. Utilising preparations abundant in isoflavones like genistein, daidzein, and biochanin A offers benefits for various conditions, including mood disorders, sedation, and alleviating hot flashes, among others.

#### 3.1.2. Physical Emulsions Parameters

The dynamic viscosity values displayed variations among the samples. Alginate samples exhibited lower dynamic viscosity than chitosan samples (Table 5). Comparing the samples, it is noticeable that the used emulsifier influenced dynamic viscosity. Incorporating β-CD as an emulsifier, in contrast to mustard extract, increased viscosity significantly. Emulsions formulated with a 2% alginate solution demonstrated statistically significant higher viscosity (samples A1M and A1B—3460.6 ± 51.2 and 4425.8 ± 78.4 mPa·s, respectively) compared to those with a 0.5% alginate solution (A2M and A2B—2882.0 ± 87.8 and 3072.3 ± 47.1 mPa·s, respectively) (Table 5). 

Notably, the chitosan sample C1P, with a polysorbate 80 emulsifier, exhibited the highest viscosity at 5456.4 ± 63.6 mPa·s (Table 5). Polysorbate as an emulsifier is much denser than β-CD, so the samples prepared using this emulsion had thicker properties. Decreasing ethanolic red clover extract concentration in formulation resulted in more viscous emulsions, while increasing concentrations and the use of β-CD compared to polysorbate 80 yield more fluid emulsions. 

**Figure 2:**
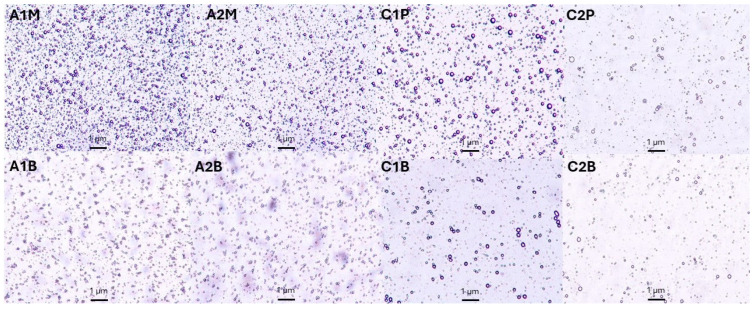
Microscopic pictures of emulsions (magnification 10×).

**Figure 3:**
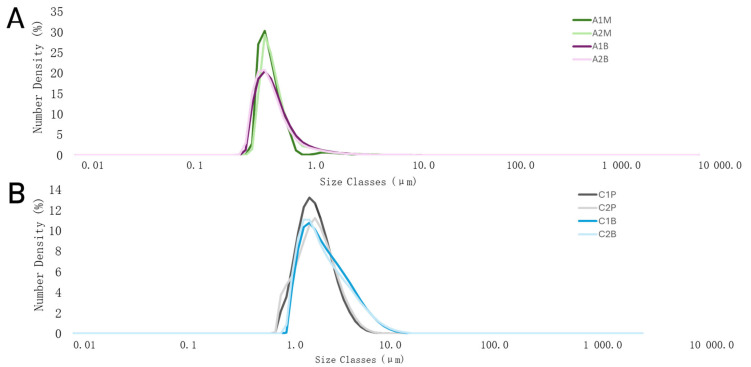
The graph of particle size distribution of (**A**)—alginate emulsions and (**B**)—chitosan emulsions. The results are mean values (*n* = 3). Sample meanings are provided in Table 3.

Precise particle size control in emulsions is crucial for improving stability, bioavailability, and sensory qualities in the pharmaceutical and food industries, leading to better formulations and consumer experiences. Microemulsions, with their smaller droplet sizes, are particularly effective in enhancing drug delivery and improving the texture and appearance. Particle size varies based on the application. Macroemulsions have larger droplets the size of 1–100 µm, while microemulsions are 100 to 1000 nm [43,44,45]. In this study, oil droplet size was evaluated in the emulsions. The percentiles D10, D50, and D90 indicate the size for which 10, 50, and 90% of the particles are equal to or less, respectively (Table 5). Figure 2 and Figure 3 show the particle size distribution for eighth emulsions in the microscope view and in a graph. The alginate emulsion droplets range from 0.3 to a maximum of 5.6 μm in size (Figure 3). 

The emulsions’ microscopic view also shows that the droplet sizes in alginate samples vary. The particle size where the cumulative distribution is 50% is the median droplet diameter. The emulsions with alginate had 50% of the particles under 0.589 μm, compared to chitosan emulsions, which was 2.64 μm, which was much higher. The chitosan emulsion droplets range from 0.8 to a maximum of 18.7 μm in size (Figure 3). A similar view can also be observed in Figure 2; alginate samples droplet sizes were smaller than chitosan. The chitosan emulsion droplet sizes were significantly higher compared to alginate emulsions (Figure 3). 

In this study, the emulsions prepared with alginate, characterised by droplet sizes ranging from 0.3 to 5.6 μm, can be classified as microemulsions. It is essential to note that microemulsions are often defined not just by their particle size (100 to 1000 nm) but also by their thermodynamic stability. This stability arises from the spontaneous formation of droplets, minimising the system’s free energy without requiring external energy for emulsification. In contrast, the chitosan-based emulsions, with droplet sizes from 0.8 to 18.7 μm, align more closely with macroemulsions, which are typically kinetically stable rather than thermodynamically stable. This distinction underscores the inherent stability advantages of microemulsions, which may offer significant benefits in applications requiring long-term stability without phase separation [46].

In this study, the oil was dispersed within a gel and water phase. Water has a refractive index of 1.33. This index is notably similar to those of alginate and chitosan solutions (ranging from 1.33 to 1.35) [47,48,49]. So, the water refractive index was used for the research to determine oil sizes. This close refractive alignment is crucial as it ensures the compatibility of the emulsion. Chitosan and alginate play integral yet distinct roles in the stabilization of emulsions. Chitosan, a cationic biopolymer, contributes to emulsion stability by forming a protective barrier around oil droplets, which prevents them from coalescing. Its positive charge effectively interacts with the negatively charged components of the oil, enhancing the emulsion’s stability [38]. On the other hand, alginate, an anionic biopolymer, stabilizes emulsions by increasing the viscosity of the aqueous phase and forming a gel matrix that traps oil droplets. This prevents them from merging and settling, thereby maintaining the uniformity and consistency of the emulsion [50]. Each biopolymer’s compatibility with the aqueous phase underpins its effectiveness in emulsion stabilization, highlighting the importance of selecting appropriate stabilizers based on their charge and interaction characteristics with the dispersed phase.

Uniformity in droplet size distribution is a critical factor contributing to the consistency and quality of emulsion-based products. Uniformity is a measure of absolute deviation from the median; comparing the uniformity of the same formulation samples but using different alginic concentrations, it is clear that sample uniformity was not the same but statistically insignificant (*p* > 0.05). Therefore, alginate concentration did not influence oil particle size. Using β-CD as an emulsifier, emulsions had higher uniformity but lower density than using mustard extract. In chitosan samples, the uniformity was almost the same. It was the same in both polysorbate samples. In the samples with β-CD, uniformity was similar. Uniform particle size can affect not just the aesthetic appeal of products but also the effectiveness of active ingredient delivery. If the uniformity is up 0.3, it might still be acceptable to consider an emulsion as having reasonably uniform droplet sizes, as can be said about all the samples in this research.

### 3.2. Microcapsules Formation

#### 3.2.1. Physical Parameters of Microcapsules

Following the evaluation of emulsion stability, microcapsule formation using the emulsions was explored. The transition from stable emulsions to robust microcapsules marks a critical step towards achieving the goal of enhancing the delivery and efficacy of bioactive compounds. The microcapsules’ physical parameters, including size, water absorption, morphology, and in vitro release profiles of phenols and isoflavones genistein daidzein and biochanin A are discussed below, illustrating the impact of our selected emulsifiers on improving bioactive compound encapsulation.

**Figure 4:**
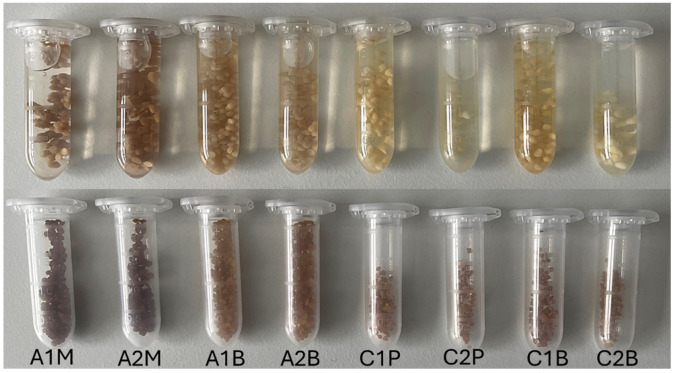
Microscopic pictures of microcapsules. On the top row are freshly prepared microcapsules in the preparation solution, and on the bottom are dried microcapsules. Sample meanings are provided in Table 3.

**Figure 5:**
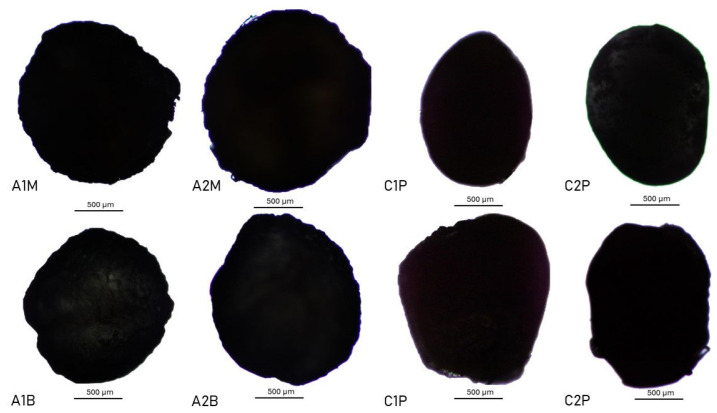
Dried microcapsule’s microscopic view via light microscope (100×). Sample meanings are provided in Table 3.

All microcapsules formed were spherical in shape and exhibited various shades of brown colouration. When placed in water, some appeared slightly yellowish (Figure 4 and Figure 5). 

Alginate microcapsules exhibited greater uniformity compared to those formed with chitosan. Microcapsules containing mustard extract appeared darker due to the extract’s light brown colouration, accompanied by a subtle mustard aroma. Notably, when β-CD was employed as an emulsifier, microcapsules exhibited reduced aggregation and achieved a more consistent morphology, contrasting with those formed using mustard extract as an emulsifier. When extruding alginate microcapsules, it was found that the optimal height from the needle to the solution surface was 20 cm, with minimal effect observed upon further increases. However, the pumping speed proved to be more critical, as exceeding 0.5 mL/min led to irregular capsule shapes.

When producing chitosan capsules at elevated heights, the formation of “tails” was observed, while higher emulsion ejection rates failed to yield uniform capsules. Due to the thicker consistency of chitosan emulsions, a lower pumping speed was necessary. Although various heights and speeds were tested, the methods outlined in Section 2.4 consistently yielded the most uniform microcapsules (Figure 5). Using polysorbate 80 as an emulsifier created more round microcapsules compared to β-CD. 

When comparing chitosan and alginate capsules, it was observed that alginate microcapsules appeared rounder and exhibited a similar structure. During extrusion, chitosan microcapsules tended to form longer shapes, yet after complete water evaporation, they appeared smaller compared to alginate microcapsules (Figure 4 and Figure 5). 

The sizes of freshly prepared microcapsules varied depending on alginate concentration (Table 6). Microcapsules with lower alginate concentrations were bigger, but their swelling capacity was lower compared with microcapsules with higher alginate concentrations. Microcapsules with mustard extract were bigger compared to microcapsules with β-CD. β-CD, composed of seven glucose units, features a unique structure with a hydrophobic cavity and a hydrophilic exterior. This enables it to form inclusion complexes with hydrophobic compounds like oils or lipids, often found in emulsions [51]. By encapsulating these hydrophobic components, β-CD improves their stability and dispersibility in water-based systems, resulting in smaller microcapsules due to enhanced emulsion stabilisation. The capsules underwent drying and shrinkage. By the end of the day, their weight remained constant, indicating stability. The correlation between size and weight remained consistent. Notably, the largest capsules measured were A2M, prepared with alginate and mustard extract, measuring 1.74 ± 0.06 mm (Table 6).

In chitosan microcapsules, the use of polysorbate 80 yielded smaller microcapsules compared with β-CD. The study by P. Eslami (2017) et al. supported our findings, indicating that the use of β-CD significantly increased microcapsule size compared to polysorbate 80. Additionally, it was noted that adjusting the amount of β-CD polysorbate 80 in the formulation had a significant impact on both encapsulation efficiency and particle size [52]. In chitosan microcapsules with β-CD, the swelling capacity was opposite that of the alginate-based samples: larger microcapsules exhibited higher water retention. The same trend was observed in microcapsules with polysorbate 80 as well, where larger microcapsules demonstrated a higher swelling capacity. Polysorbate 80 creates a protective layer that stops water evaporation, whereas chitosan increases the viscosity and thickness of the microcapsule wall, thereby diminishing water retention [26,53]. Like alginate microcapsules, those made of chitosan also experienced shrinkage. Among them, the smallest was the chitosan microcapsules C1P, incorporating polysorbate 80, measuring 1.22 ± 0.08 mm (Table 6). 

The alginate and chitosan samples experienced significant shrinkage, reducing by more than 50% of their original sizes. Notably, chitosan microcapsules containing polysorbate 80 exhibited the least shrinkage due to their lower water content (C1P~53.70% and C2P~55.70%). Likewise, samples containing β-CD showed a comparable reduction of approximately 75%. Alginate samples prepared with mustard experienced shrinkage of around 61.56% and 68.25% for A1M and A2M, respectively (Table 6). When comparing alginate and chitosan microcapsules, a decrease in alginate concentration coupled with the use of β-CD leads to an increase in size. This is because fewer alginate molecules are available for gel formation, enabling greater expansion. In contrast, increasing extract concentration in chitosan microcapsules and incorporating β-CD resulted in smaller microcapsules. This is due to the reduced availability of chitosan for gel formation in ethanolic extract and additional phenols, leading to smaller microcapsules, an effect that is further enhanced by the presence of β-CD.

Alginate microcapsules were much stronger than chitosan. A1M sample had the highest firmness—4400.01 ± 159.66 g/The microcapsules prepared from emulsions with higher alginate concentrations were harder, and the use of mustard extract also influenced the firmness (Table 6). In the samples of alginate microcapsules, smaller microcapsules (A1M; A1B) showed higher firmness compared to larger ones (A2M; A2B). This was particularly noticeable in the larger capsules with lower alginate concentrations, leading to reduced water capacity (Table 6).

The firmness of the chitosan samples was significantly lower compared to that of the alginate samples. The stronger capsules were the ones with lower extract concentrations. Using emulsifiers in the C1 formulation has not considerably improved the firmness. However, using β-CD in formulation C2 (C2B) yielded higher firmness compared to polysorbate 80 sample (C2P). 

#### 3.2.2. In Vitro Release of Bioactive Compounds from Microcapsules

**Figure 6:**
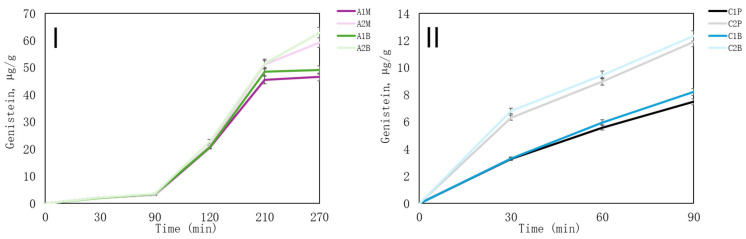
In vitro release of genistein from microcapsules. (**I**)—the release of microcapsules from alginate; (**II**)—the release of microcapsules from chitosan. The results are mean values (*n* = 3). Sample meanings are provided in Table 3.

**Figure 7:**
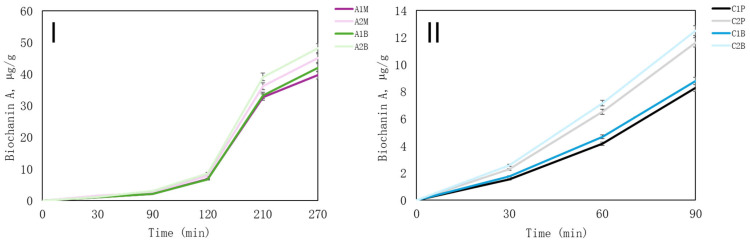
In vitro release of biochanin A from microcapsules. (**I**)—the release of microcapsules from alginate; (**II**)—the release of microcapsules from chitosan. The results are mean values (*n* = 3). Sample meanings are provided in Table 3.

**Figure 8:**
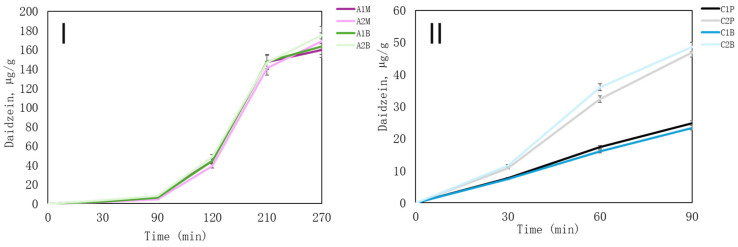
In vitro release of daidzein from microcapsules. (**I**)—the release of microcapsules from alginate; (**II**)—the release of microcapsules from chitosan. The results are mean values (*n* = 3). Sample meanings are provided in Table 3.

In this research, in vitro release from all the capsules was investigated, and total phenolic compounds (Table 7) and isoflavones—daidzein, genistein and biochanin A—were determined in the media (Figure 6, Figure 7 and Figure 8). Chitosan microcapsules were fully solubilised in the stomach media; therefore, after 90 min, they were not continuously put through the intestine media. However, alginate microcapsules are soluble only in the alkaline media, so the active compounds were released in the gut media (Table 6). Nevertheless, after 270 min of in vitro release (150 min in gut media), not all alginate microcapsules entirely disintegrated. A1 formulation microcapsules were still intact, even though most of the capsules were dissolved. These samples had the thicker shells of the increased alginate concentration in the formulation.

The bioactive compounds from microcapsules were released gradually. Most of the phenolic compounds were released from the microcapsules, but some extract losses occurred during the microencapsulation process. The highest phenolic content of alginate microcapsules was released after 210 min. The formulation A2 (when the alginate concentration was 0.5%) solubilised faster than the A1 formulation with either β-CD or mustard extract (Table 7). 

Chitosan microcapsules also gradually solubilised the C2 formulation, releasing almost all the active compounds. As in the alginate microcapsules, active compounds were lost because of the microencapsulation process and washing off of the extract. Nevertheless, by combining microcapsules of chitosan and alginate, it would be possible to obtain a product that can be soluble in the stomach and intestine for the best effect.

The release of three isoflavones—genistein, daidzein, and biochanin A—from microcapsules was assessed. Figure 6 illustrates the release of genistein from both alginate and chitosan microcapsules. Studies on portal vein plasma levels have indicated that genistein exhibits higher bioavailability in its aglycon form compared to its glycoside counterpart. Specifically, genistein is only partially absorbed in its glycosidic form [54]. Because of this reason, red clover extract was prepared by combining ultrasound processing and heat reflux. This extraction helps yield genistein instead of genistin. As mentioned before, after 270 min of in vitro release, only samples A1M and A1B did not completely dissolve, suggesting that not all of the active compounds were released. Most of the genistein was released in the sample A2B—62.85 ± 1.89 µg/g. The lowest concentration of genistein was released from sample A1M—46.57 ± 1.4 µg/g (Figure 6I). Genistein was released gradually, with formulation A2, particularly with β-CD, yielding superior results. Cyclodextrins (CDs) facilitate the delivery of poorly water-soluble and chemically unstable drugs to the body, thereby enhancing drug solubility when incorporated into microcapsules [55]. Genistein is mainly absorbed in the gastrointestinal tract, especially in the small intestine, where nutrient absorption primarily occurs. Its excellent bioavailability ensures efficient absorption throughout the gastrointestinal tract [56].

A similar trend was observed in chitosan microcapsules, where the incorporation of β-CD as an emulsifier resulted in improved genistein release. Specifically, formulation C2 exhibited higher isoflavone release, but within this formulation, the sample with β-CD (C2B) demonstrated greater genistein release compared to C2P with polysorbate 80 (Figure 6II).

A similar release trend was observed for daidzein and biochanin A, mirroring that of genistein. Biochanin A exhibits rapid absorption in the gut owing to its favourable permeability and lipophilic properties. Despite this, biochanin A is noted for its poor bioavailability [57]. The release of biochanin A is shown in Figure 7.

As alginate microcapsules are not soluble in acidic conditions, the release of isoflavones in stomach media is limited. The isoflavones that were released may have remained on the surface after microencapsulation. Most of the biochanin was released after 210 min in vitro (Figure 7I). 

All chitosan samples were completely dissolved in stomach media. Among them, C2 formulations exhibited the highest release rates (Figure 7II). However, the concentrations of released biochanin A were relatively low. This may be attributed to its poor solubility, despite attempts to enhance it with the excipients used in the microcapsules.

Most of all, isoflavones daidzein was released in the highest concentrations in chitosan and alginate microcapsules (Figure 8I,II). Because of its chemical structure, daidzein as genistein and biochanin A exhibit low solubility in both water and lipids. Consequently, it is primarily absorbed in the intestinal tract following oral administration and readily undergoes metabolism, forming glucuronic acid or sulfuric acid conjugates [58].

Formulations A2 and C2, especially when formed with β-CD, demonstrate considerable potential for further refinement and advancement as proficient delivery systems for bioactive compounds. Moreover, incorporating mustard into alginate-based samples can enhance microcapsules by enriching them with additional flavonoids and phenolic compounds. Determining the in vitro release of isoflavones provides valuable insights into their behaviour in formulated products, aiding in the development of effective delivery systems and therapeutic strategies. 

## 4. Conclusions

In conclusion, this study demonstrates the effectiveness of mustard extract and β-CD as emulsifiers in forming stable microcapsules, with alginate showing high stability. The choice of emulsifier significantly influences microcapsule morphology, emphasising the need for careful formulation design. Alginate microcapsules exhibit controlled release in both formulations, while chitosan microcapsules dissolve in stomach media, suggesting targeted delivery potential. The A2 and C2 formulations showed the most potential of all the samples with optimal swelling, release and firmness. Incorporating β-CD enhances bioactive compound release, offering opportunities for improving delivery systems in both gelificators samples, whereas mustard with alginate could additionally enrich the samples with phenols. Future research should focus on refining formulations for enhanced efficiency and kinetics. The use of alginate and chitosan microcapsules in a single pill holds promise for versatile delivery systems. These microcapsules, soluble in both stomach and intestines, containing phenols and isoflavones from red clover, could offer enhanced bioavailability and targeted delivery, benefiting various applications in health supplements, foods, pharmaceuticals, and drug delivery systems. Future investigations should focus on the clinical implications of these delivery systems, exploring their safety, efficacy, and potential benefits for human health.

## Figures and Tables

**Table 1:** Quantities of the components used in emulsions formulations that were prepared using alginate.

Number of Emulsions	Alginate, %	Extract, %	Mustard Extract, %	β-CD Extract, %	Oil, %	Xanthan, %	Water, %
1 *	1	35	5	-	5	5	49
2 *	0.25	35	5	-	5	5	49.75
3 *	1	35	-	5	5	5	49
4 *	0.25	35	-	5	5	5	49.75
5	1	30	5	-	10	5	49
6	0.25	30	5	-	10	5	49.75
7	1	30	-	5	10	5	49
8	0.25	30	-	5	10	5	49.75
9	1	30	5	-	5	10	49
10	0.25	30	5	-	5	10	49.75
11	1	30	-	5	5	10	49
12	0.25	30	-	5	5	10	49.75
13	1	35	5	-	2.5	7.5	49
14	0.25	35	5	-	2.5	7.5	49.75
15	1	35	-	5	2.5	7.5	49
16	0.25	35	-	5	2.5	7.5	49.75

* Means that these formulations were the most stable.

**Table 2:** Emulsions formulations that were prepared using chitosan.

Number of Emulsions	Chitosan, %	Extract, %	Polysorbate 80, %	β-CD, %	Oil, %	Acidified Water, %
1	0.7	40	1	-	3	34.3
2	0.7	40	-	1	3	34.3
3	0.92	50	1	-	3	45.08
4	0.92	50	-	1	3	45.08
5	0.9	51	1	-	3	44.1
6	0.9	51	-	1	3	44.1
7 *	2.00	10	1	-	3	84.00
8 *	2.00	10	-	1	3	84.00
9 *	2.00	20	1	-	3	74.48
10 *	2.00	20	-	1	3	74.48
11	2.00	30	1	-	3	64.68
12	2.00	30	-	1	3	64.68

* Means that these formulations were the most stable.

**Table 3:** Emulsions formulations and codes of chitosan and alginate samples.

Sample Code	Alginate, %	Chitosan, %	Extract, %	Mustard Extract, %	β-CD, %	Polysorbate 80, %	Oil, %	Xanthan Gum, %	Water, %	Acidified Water, %
A1M	1	-	35	5	-	-	5	5	49	-
A2M	0.25	-	35	5	-	-	5	5	49.75	-
A1B	1	-	35	-	5	-	5	5	49	-
A2B	0.25	-	35	-	5	-	5	5	49.75	-
C1P	-	2.00	10	-	-	1	3	-	-	84.00
C2P	-	2.00	20	-	-	1	3	-	-	74.00
C1B	-	2.00	10	-	1	-	3	-	-	84.00
C2B	-	2.00	20	-	1	-	3	-	-	74.00

A, C—alginate (A) or chitosan (C) was used as a shell material; 1; 2—the concentrations of the polymers; M, B, P- indicates emulsifiers used in formulation.

**Table 4:** The total content of active compounds in the emulsions. The results are mean values (n = 3). Sample meanings are provided in Table 3.

	Total Phenolic Content, mg GA/g	Total Flavonoid Content, mg RU/g	Daidzein, µg/g	Genistein, µg/g	Biochanin A, µg/g
Red clover extract (concentrated)	102.56 ± 2.15	29.43 ± 0.36	1066.97 ± 20.45	205.77 ± 12.89	139.73 ± 7.62
Mustard extract	22.43 ± 1.21	13.97 ± 0.39	-	-	-
A1M	66.62 ± 1.56	20.62 ± 0.52	642.54 ± 21.34	119.22 ± 3.46	82.69 ± 3.66
A2M	67.62 ± 1.68	20.95 ± 0.25	638.36 ± 32.41	126.32 ± 5.33	81.20 ± 6.87
A1B	60.62 ± 1.32	18.65 ± 0.81	664.45 ± 18.22	124.47 ± 6.97	84.92 ± 5.12
A2B	61.45 ± 1.45	18.82 ± 0.95	648.92 ± 24.81	126.12 ± 4.23	79.16 ± 5.73
C1P	10.11 ± 0.19	2.47 ± 0.06	99.68 ± 6.68	18.64 ± 1.92	11.91 ± 0.75
C2P	17.98 ± 0.54	5.62 ± 0.28	206.96 ± 12.87	34.99 ± 6.98	23.78 ± 2.64
C1B	9.98 ± 0.12	2.89 ± 0.19	94.79 ± 8.82	18.33 ± 3.14	12.15 ± 0.58
C2B	18.61 ± 0.68	5.78 ± 0.22	210.46 ± 11.56	36.21 ± 5.74	24.54 ± 1.01

**Table 5:** Parameters of emulsions. The results are mean values (n = 3). Sample meanings are provided in Table 3.

Sample	Dynamic Viscosity, mPa·s	Dx(10)	Dx(50)	Dx(90)	Uniformity
A1M	3460.6 ± 51.2	0.449	0.561	0.931	0.365
A2M	2882.0 ± 87.8	0.469	0.589	0.944	0.370
A1B	4425.8 ± 78.4	0.387	0.525	0.934	0.399
A2B	3072.3 ± 47.1	0.402	0.550	0.981	0.396
C1P	5456.4 ± 63.6	1.39	2.19	3.81	0.346
C2P	4654.5 ± 81.9	1.31	2.00	3.82	0.346
C1B	5156.6 ± 58.4	1.58	2.64	3.91	0.319
C2B	4284.8 ± 65.3	1.52	2.50	3.94	0.357

**Table 6:** Microcapsules firmness and swelling parameters. The results are mean values (*n* = 3). Sample meanings are provided in Table 3.

	Firmness, g	Microcapsules Diameter (mm)		Swelling, %
		Wet	Dry	
A1M	4400.01 ± 159.66	2.58 ± 0.06	1.49 ± 0.10	135.37 ± 2.45
A2M	3138.10 ± 104.12	3.06 ± 0.03	1.74 ± 0.06	124.86 ± 1.56
A1B	3512.23 ± 51.97	2.18 ± 0.04	1.35 ± 0.07	146.11 ± 1.67
A2B	2609.90 ± 214.37	2.46 ± 0.08	1.46 ± 0.12	132.82 ± 1.11
C1P	1144.91 ± 110.29	1.79 ± 0.09	1.22 ± 0.08	268.19 ± 1.65
C2P	538.31 ± 20.32	1.97 ± 0.05	1.27 ± 0.06	310.85 ± 3.56
C1B	1266.78 ± 97.97	2.37 ± 0.05	1.32 ± 0.08	271.52 ± 2.64
C2B	960.40 ± 60.59	2.15 ± 0.02	1.26 ± 0.11	248.39 ± 1.84

**Table 7:** In vitro release of phenolic compounds from microcapsules. The results are mean values (*n* = 3). Sample meanings are provided in Table 3.

	30 min, mg GAE/g	60 min, mg GAE/g	90 min, mg GAE/g	120 min, mg GAE/g	150 min, mg GAE/g	180 min, mg GAE/g	210 min, mg GAE/g	270 min, mg GAE/g
A1M	1.99 ± 0.13	2.39 ± 0.13	2.82 ± 0.14	16.84 ± 0.16	18.32 ± 0.1	19.66 ± 0.12	20.32 ± 0.16	20.84 ± 0.29
A2M	2.31 ± 0.1	2.8 ± 0.2	3.23 ± 0.07	17.34 ± 0.05	18.26 ± 0.12	18.76 ± 0.1	22.71 ± 0.08	28.97 ± 0.14
A1B	1.79 ± 0.02	2.03 ± 0.08	3.04 ± 0.08	14.23 ± 0.1	14.88 ± 0.16	15.94 ± 0.18	17.4 ± 0.08	17.92 ± 0.06
A2B	1.6 ± 0.08	2.45 ± 0.02	3.05 ± 0.06	15.35 ± 0.12	16.98 ± 0.09	17.42 ± 0.12	18.33 ± 0.02	23.65 ± 0.09
C1P	9.2 ± 0.41	13.56 ± 0.46	14.83 ± 0.64	-	-	-	-	-
C2P	11.42 ± 0.56	16.5 ± 0.25	18.14 ± 0.19	-	-	-	-	-
C1B	7.98 ± 0.26	11.65 ± 0.2	14.52 ± 0.56	-	-	-	-	-
C2B	10.11 ± 0.18	14.65 ± 0.16	16.59 ± 0.6	-	-	-	-	-

## Data Availability

Data are contained within the article.
